# Decoding the impact of MMP1+ malignant subsets on tumor-immune interactions: insights from single-cell and spatial transcriptomics

**DOI:** 10.1038/s41420-025-02503-y

**Published:** 2025-05-20

**Authors:** Da-Ming Xu, Ling-Xiao Chen, Ting Xue, Xiao-Yu Zhuang, Li-Chao Wei, Hui Han, Miao Mo

**Affiliations:** 1 State Key Laboratory of Oncology in South China, Guangdong Provincial Clinical Research Center for Cancer, Sun Yat-sen University Cancer Center, Guangzhou, P. R. China; 2 Department of Urology, Sun Yat-sen University Cancer Center, Guangzhou, P.R. China; 3 Department of Urology, Xiangya Hospital, Central South University, Changsha, P.R. China; 4 Department of Anesthesiology, Second Affiliated Hospital of Shantou University Medical College, Shantou, P. R. China; 5 Department of Organ Transplantation, Sichuan Provincial People’s Hospital, School of Medicine, University of Electronic Science and Technology of China, Chengdu, P.R. China

**Keywords:** Prognostic markers, Cancer microenvironment

## Abstract

Matrix metalloproteinase 1 plays a pivotal role in tumor biology and immune modulation through its enzymatic remodeling of the extracellular matrix, facilitating tumor progression. In this study, we utilized large-scale single-cell RNA sequencing and spatial transcriptomics to investigate MMP1 expression, its cellular localization, and its impact on tumor progression and immune modulation. Our findings reveal that MMP1 expression is elevated in various tumor types and is strongly correlated with metastatic potential. High MMP1 expression was associated with increased activity in epithelial–mesenchymal transition signaling and TNFα/NF-κB pathways, which are known to promote tumor progression. Furthermore, MMP1+ malignant cells exhibited significant interactions with immune cells, particularly macrophages and CD8+ T cells. MMP1 expression correlated with enhanced macrophage infiltration and impaired CD8+ T-cell function, contributing to an immunosuppressive tumor microenvironment. Notably, the CXCL16-CXCR6 and ANXA1-FPR3 signaling axes were identified as key mediators of these interactions. Inhibition of MMP1 in vitro demonstrated reduced cell invasion, stemness, and proliferation, while increasing reactive oxygen species levels and promoting apoptosis. Our findings position MMP1 as a key player in the “tumor-immune” vicious cycle and a promising therapeutic target to enhance anti-tumor responses and improve patient outcomes.

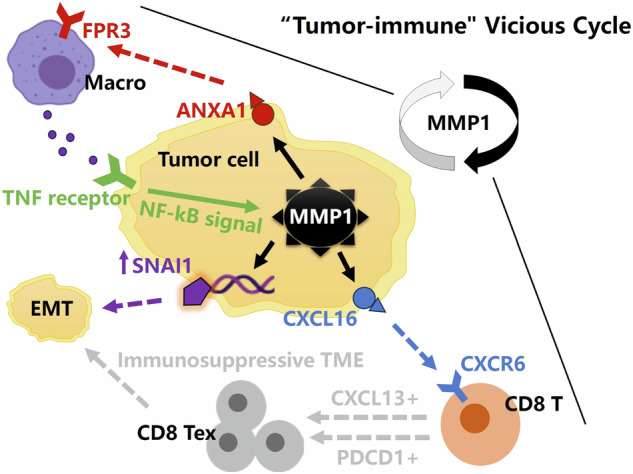

## Introduction

Matrix metalloproteinase 1 (MMP1) plays a pivotal role in tumor biology and immune regulation due to its critical function in extracellular matrix (ECM) remodeling [1, 2]. Specifically, MMP1 primarily targets type I collagen, a fundamental structural protein in the ECM, for degradation [3]. This enzymatic activity is central to various physiological and pathological processes, including tissue remodeling, wound healing, and tumor progression [4]. In tumor, MMP1’s ability to degrade ECM components facilitates tumor cell invasion and metastasis by breaking down physical barriers within the ECM [5]. This process enables the dissemination of tumor cells into surrounding tissues and distant organs, contributing to both tumor spread and a poorer prognosis.

In addition to its structural role, MMP1 significantly influences tumor-immune interactions by modulating immune cell infiltration and positioning within the tumor microenvironment (TME) [6]. Through ECM remodeling, MMP1 alters the accessibility and functionality of immune cells, potentially affecting their ability to mount effective anti-tumor responses. Notably, MMP1 may promote immune evasion by creating a TME that favors tumor growth and survival. For instance, the enzyme can enhance tumor progression by supporting immunosuppressive mechanisms that allow tumor cells to escape immune surveillance [7]. Understanding the multifaceted roles of MMP1, particularly its dual impact on ECM remodeling and immune modulation, is crucial for uncovering mechanisms of tumor progression and immune escape. Such insights are essential for developing targeted therapeutic strategies aimed at inhibiting MMP1 and its associated malignant subgroups.

In this study, we leveraged large-scale single-cell RNA sequencing (scRNA-seq) and spatial transcriptomic (ST) analyses to gain deeper insights into the role of MMP1 in tumors. These advanced technologies provided a detailed understanding of MMP1 expression patterns and their spatial distribution within the tumor microenvironment. Through these analyses, we elucidated how MMP1 contributes to the malignant behavior of tumor cells and their interactions with immune cells. Additionally, our functional assays for MMP1 inhibition demonstrated its promising therapeutic potential in combating malignancies. By targeting MMP1, we aim to suppress tumor progression and enhance the efficacy of existing therapeutic strategies. Collectively, this research underscores the critical role of MMP1 in tumor biology and immune modulation, offering valuable insights that could inform the development of innovative treatments and improve outcomes for tumor patients.

## Results

### Comprehensive analysis of MMP1 gene expression, localization, and function in tumors

**Fig. 1:**
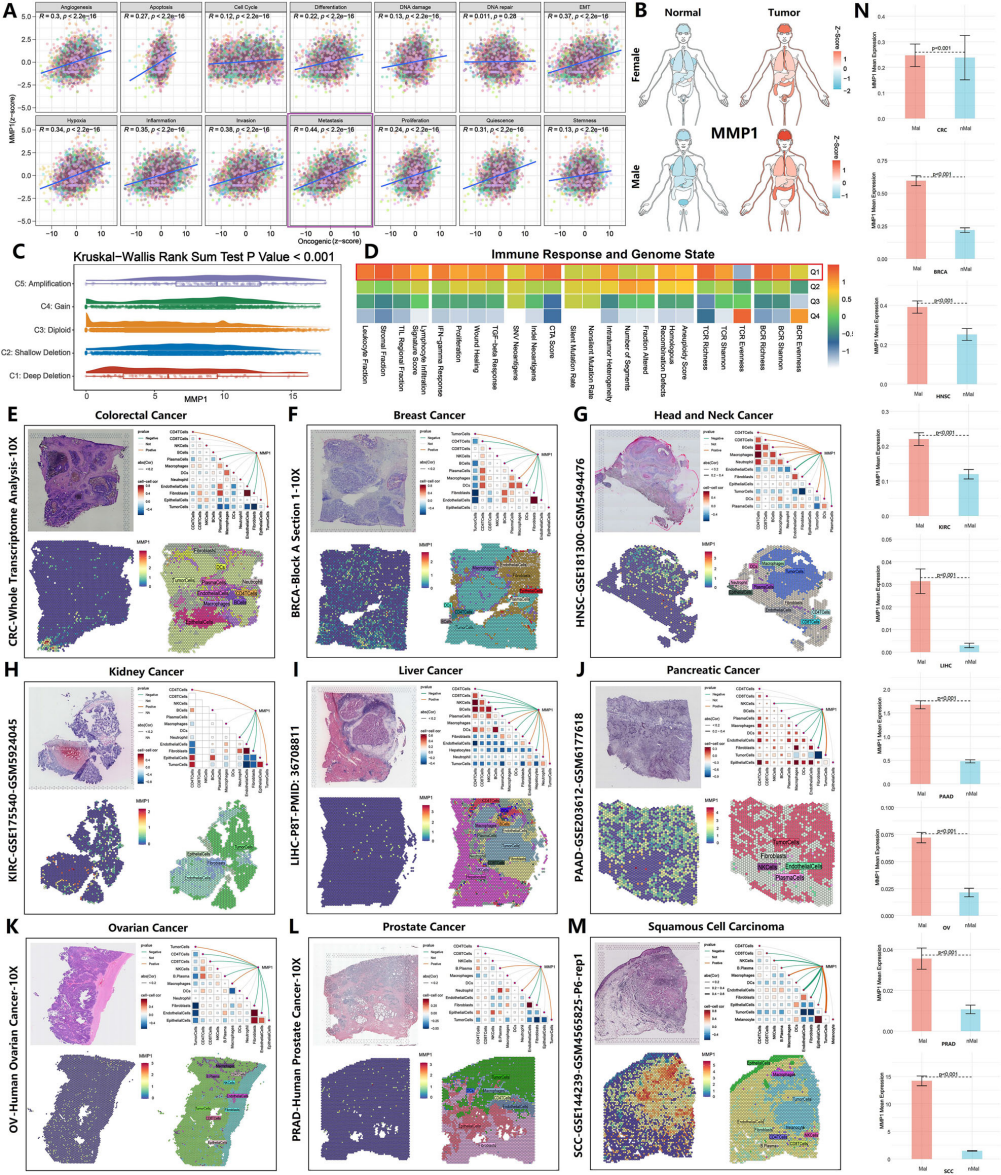
MMP1 expression and its correlation with tumor metastasis in pan-cancer analysis. **A** MMP1 expression levels across multiple tumor types demonstrate a strong positive correlation with tumor metastasis. **B** Comparative analysis of MMP1 expression between normal and tumor tissues, showing significant upregulation in tumors. **C** Correlation between MMP1 expression and copy number alterations, indicating a progressive increase from homozygous deletions to high-copy number amplifications. **D** Immune response analysis of tumors with MMP1 overexpression, revealing a more robust immune activation in high MMP1 groups. **E**–**M** Spatial transcriptomic analysis of MMP1 expression in tumor microenvironments, highlighting its localization in specific malignant cell subpopulations and its strong association with tumor cell clusters. **N** The expression differences of MMP1 between the malignant (Mal) and non-malignant (nMal) regions.

Across pan-cancer analyses, MMP1 expression showed the strongest positive correlation with tumor cell metastasis scores and was significantly upregulated in the majority of tumors (Fig. 1A, B; Fig. S1). As homozygous deletions transitioned to high-copy number amplifications, MMP1 expression levels exhibited a consistent upward trend. Notably, tumors with MMP1 overexpression displayed a more robust immune response (Fig. 1C, D). At ST resolution, MMP1 expression demonstrated a similar localization pattern with malignancy, showing a strong positive correlation with tumor cells. High-expression regions were predominantly concentrated in a small subset of malignant cell populations (Fig. 1E–M). Furthermore, we defined microregions with a malignant cell proportion greater than 0 as malignant regions (Mal) and microregions with a malignant cell proportion of 0 as non-malignant regions (nMal) (Fig. S2). Our analysis showed that MMP1 expression was significantly higher in the Mal regions compared to the nMal regions (Fig. 1N). These findings suggested that MMP1+ malignant cell subset likely represented a pre-metastatic population and served as a key driver of tumor cell metastasis.

### Enhanced epithelial–mesenchymal transition (EMT) and TNFα/NF-κB signaling pathway activity in tumors with elevated MMP1 expression

**Fig. 2:**
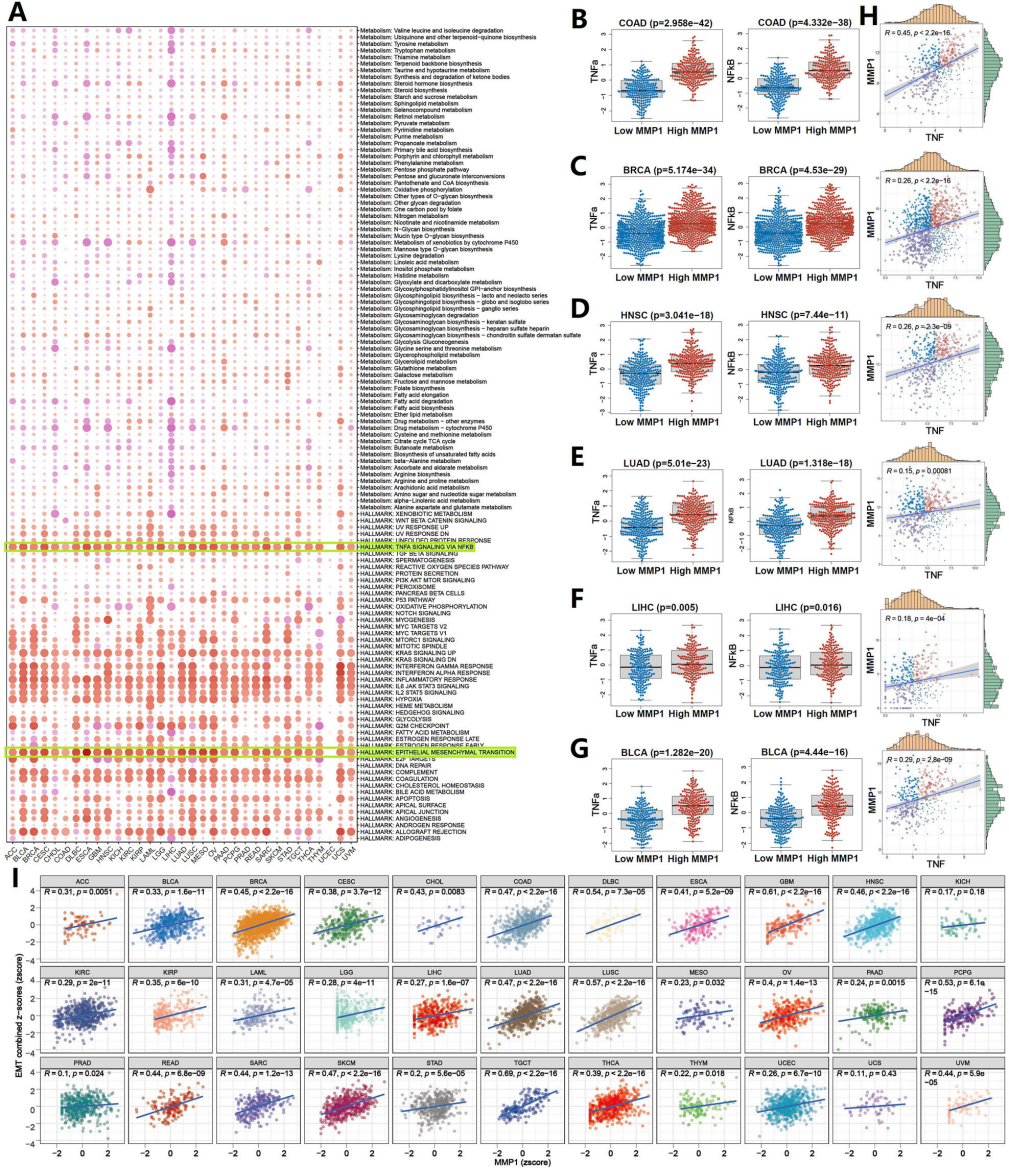
Role of MMP1 in tumorigenesis and its association with key signaling pathways. **A** Gene enrichment analysis based on the Hallmark and KEGG metabolic gene sets shows significant enrichment of the EMT and TNFα/NF-κB signaling pathways in the high MMP1 expression group. **B**–**G** PROGENy algorithm results validating the upregulation of TNFα and NF-κB signaling pathways in tumors with high MMP1 expression. **H** Positive correlation between MMP1 expression and TNF levels across multiple tumor types, indicating a potential regulatory relationship. **I** Positive correlation between MMP1 expression and EMT score.

Abnormal signaling pathways played a crucial role in tumorigenesis. To explore this, we performed gene enrichment analysis using the Hallmark and KEGG. Our results revealed that high MMP1 expression was significantly associated with enrichment in the EMT and TNFα/NF-κB signaling pathways (Figs. 2A and S3). Validation with the PROGENy algorithm confirmed a marked upregulation of both TNFα and NF-κB signaling pathways in the high MMP1 expression group (Fig. 2B–G). Previous studies have shown that TNFα/NF-κB signaling pathways can upregulate the expression of matrix metalloproteinases [8, 9]. Consistently, we observed a significant positive correlation between MMP1 and TNF expression across multiple tumors (Figs. 2H and S4). Additionally, the expression of the MMP1 gene exhibited a significant positive correlation with the EMT score, as indicated by the CancerSEA database (Fig. 2I). These findings suggest that the EMT and TNFα/NF-κB signaling pathways may drive the upregulation of MMP1 in tumors.

### Interactions between MMP1+ malignant cells, macrophages, and CD8+ T cells

**Fig. 3:**
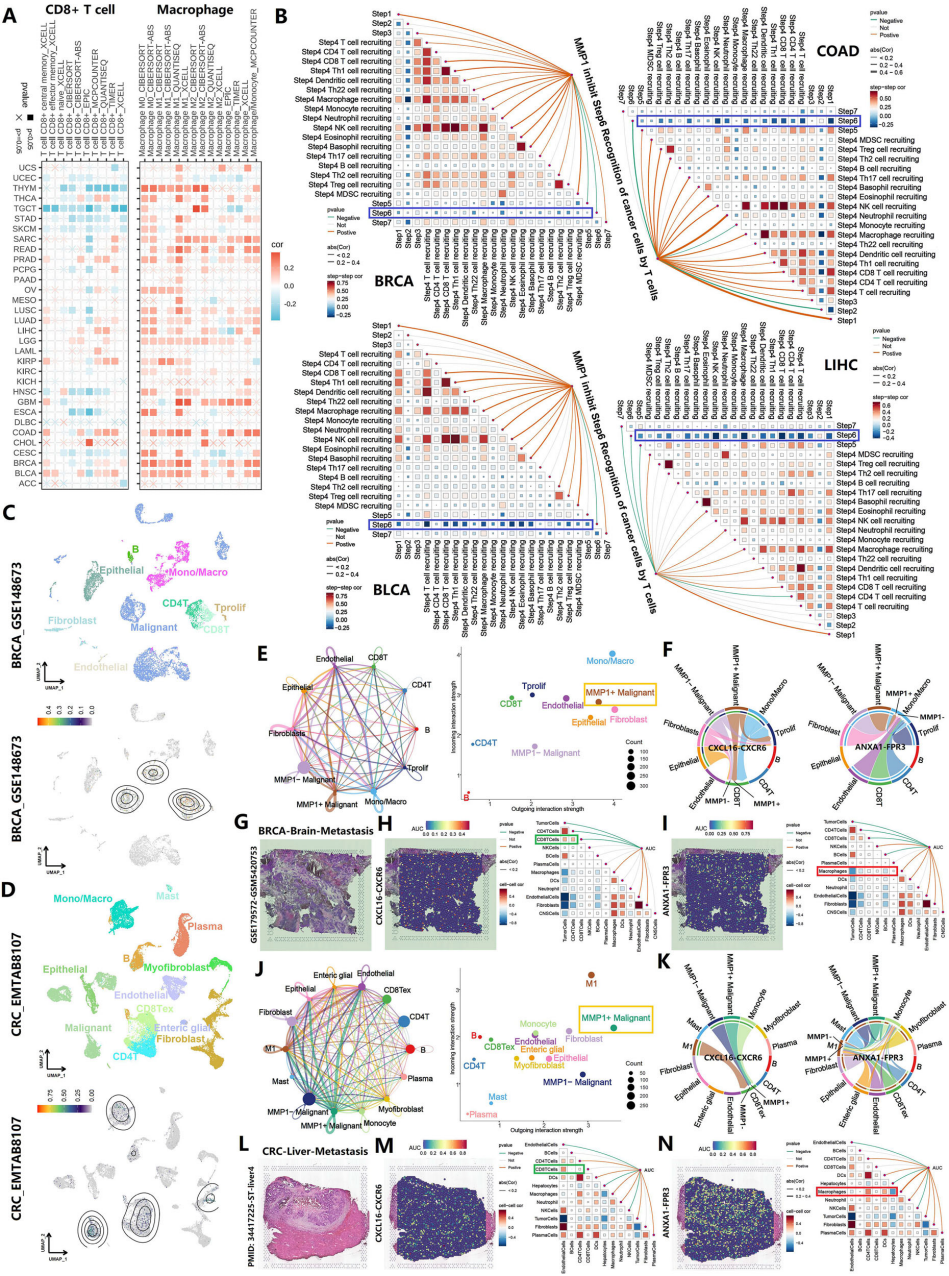
MMP1’s role in immune infiltration, immune cycle disruption, and ligand-receptor signaling in the tumor microenvironment. **A** Immune infiltration analysis using multiple algorithms revealed that MMP1 promotes increased macrophage infiltration while reducing CD8+ T-cell infiltration. **B** Immune cycle analysis suggested that MMP1 impaired T-cell function across four common tumor types. **C**, **D** Single-cell analyses of breast cancer (BRCA, GSE148673) and colorectal cancer (CRC, EMTAB8107) demonstrated that MMP1 expression is predominantly localized in malignant cells, macrophages, and T cells. **E**, **J** Cell communication analysis indicates that MMP1+ malignant cells exhibit stronger outgoing signals compared to MMP1- malignant cells. **F**, **K** Ligand-receptor pathway analyses showed that MMP1+ malignant cells exerted strong regulatory effects on CD8+ T-cell activity through the CXCL16-CXCR6 signaling axis and on macrophages through the ANXA1-FPR3 signaling axis. **G**, **L** ST analysis of brain metastases from breast cancer and liver metastases from colorectal cancer. **H**, **M** The CXCL16-CXCR6 axis’s suppressive effect on CD8+ T cells. **I**, **N** The ANXA1-FPR3 axis’s enhancement of macrophage activity.

Immune infiltration analysis using multiple algorithms revealed that MMP1 promotes increased macrophage infiltration while reducing CD8+ T-cell infiltration (Fig. 3A). The correlation analysis of MMP1 immune signatures suggested that MMP1 may play an important role in immune regulation (Fig. S5). Furthermore, MMP1 expression was closely associated with the activation of numerous immune-related genes (Fig. S6). Analysis of the immune cycle indicated that MMP1 impairs T-cell function across four common tumor types (Fig. 3B). Single-cell analyses of breast cancer (BRCA, GSE148673) and colorectal cancer (CRC, EMTAB8107) confirmed that MMP1 expression is predominantly localized to malignant cells, macrophages, and T cells (Fig. 3C, D). Interestingly, MMP1+ malignant cells exhibited stronger outgoing signals in cell communication than their MMP1− counterparts (Fig. 3E, J). Ligand-receptor pathway analysis in BRCA revealed that the regulatory effects of MMP1+ malignant cells on CD8+ T cells via CXCL16-CXCR6 signaling axis and macrophages via ANXA1-FPR3 signaling axis were significantly stronger than those of MMP1− cells (Fig. 3F). Similar findings were observed in CRC (Fig. 3K). ST analysis of brain metastases from breast cancer (Fig. 3G) and liver metastases from CRC (Fig. 3L) suggested that the CXCL16-CXCR6 signaling axis did not promote CD8+ T-cell activation or even exerted a suppressive effect (Fig. 3H, M), while the ANXA1-FPR3 signaling axis may enhance macrophage activity (Fig. 3I, N).

### MMP1+ malignant cells’ role in the “tumor-immune” vicious cycle: insights into CXCL16-CXCR6 and ANXA1-FPR3 pathways

**Fig. 4:**
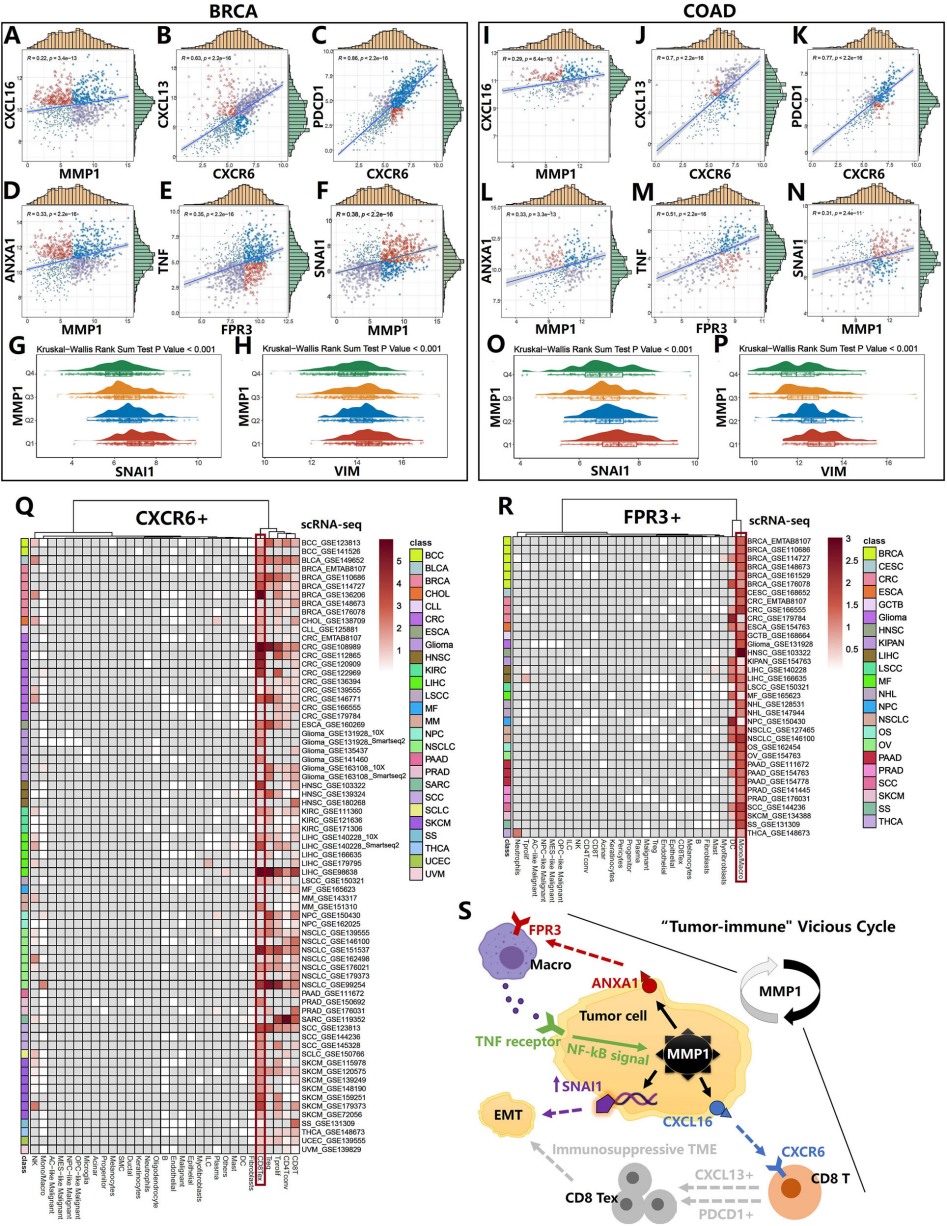
MMP1 activation of CXCL16-CXCR6 and ANXA1-FPR3 pathways in tumor progression and immune modulation. **A**–**F**, **I**–**N** MMP1 activation of the CXCL16-CXCR6 and ANXA1-FPR3 pathways upregulated the T-cell dysfunction markers CXCL13 and PDCD1 while inducing macrophages to release TNF. **G**, **H**, **O**, **P** Overexpression of MMP1 led to abnormal activation of EMT markers SNAI1 and VIM. **Q**, **R** Pan-cancer single-cell analyses revealed a strong relationship between CXCR6 and T-cell exhaustion, indicating its role in tumor-immune evasion, while the strong association between FPR3 and macrophages highlighted its importance in shaping the tumor microenvironment. **S** These findings reinforced the hypothesis that MMP1 drived tumor progression and immune modulation through its involvement in the CXCL16-CXCR6 and ANXA1-FPR3 pathways.

The CXCL16-CXCR6 and ANXA1-FPR3 signaling pathways were identified as key mechanisms implicated in various tumor types (Fig. S7). Based on gene expression correlation analyses and supporting evidence from previous studies [10, 11], we hypothesized that MMP1 activates the CXCL16-CXCR6 axis, leading to the upregulation of T-cell dysfunction markers CXCL13 and PDCD1. Concurrently, MMP1 activation of the ANXA1-FPR3 axis induced macrophages to release TNF (Fig. 4A–F, I–N). Additionally, MMP1 overexpression was associated with the abnormal activation of EMT markers SNAI1 and VIM, further supporting its role in promoting EMT (Fig. 4G, H, O, P). Pan-cancer analyses using large-scale single-cell data highlighted a close relationship between CXCR6 expression and T-cell exhaustion, suggesting a critical role for CXCR6 in tumor-immune evasion (Fig. 4Q). Similarly, the strong association of FPR3 with macrophages emphasized its essential role in shaping the tumor microenvironment (Fig. 4R). These findings not only reinforced our hypotheses but also provided a foundation for deeper insights into the functional roles of these pathways across various tumor types (Fig. 4S).

### Prediction of upstream positive and negative regulatory transcription factors for MMP1

**Fig. 5:**
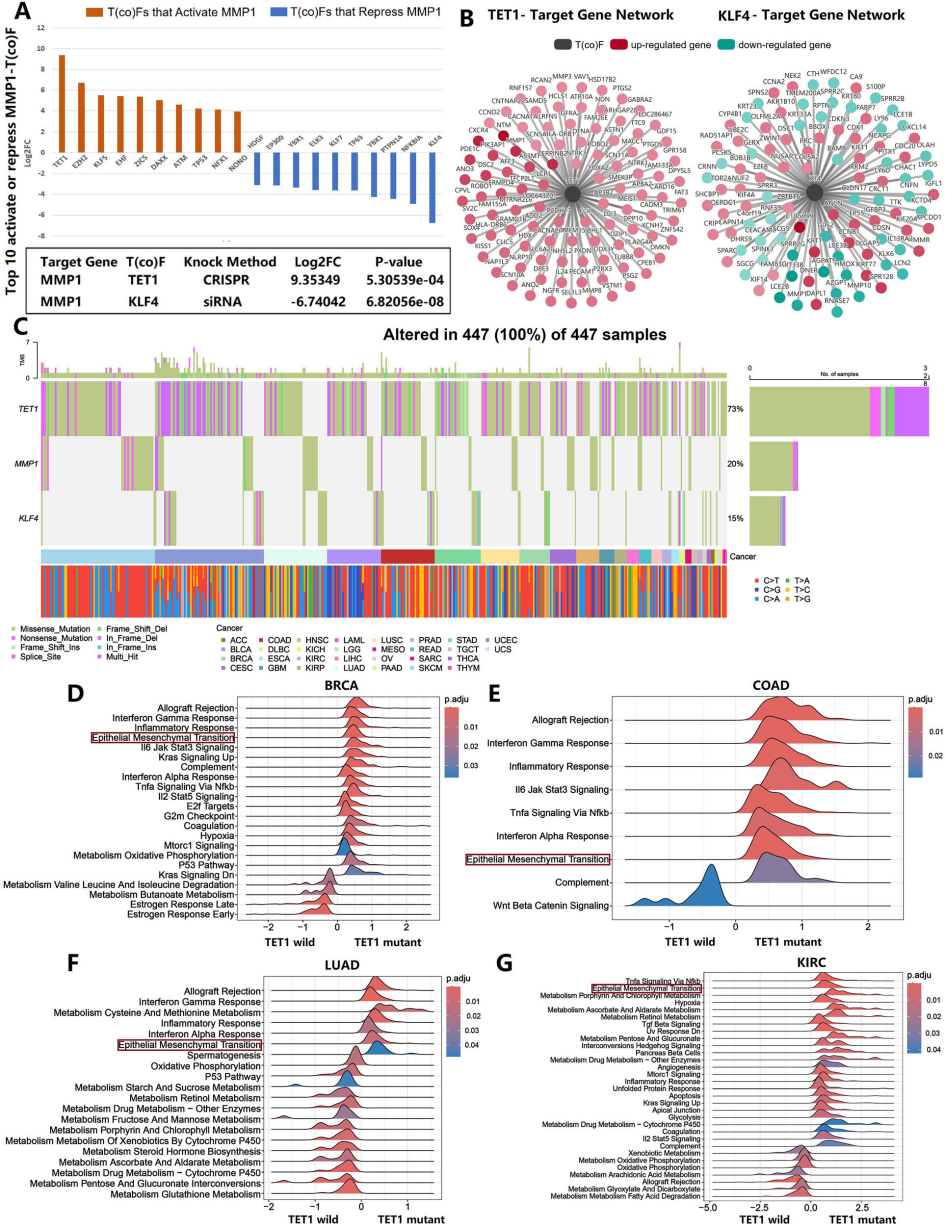
TET1 and KLF4 as key transcriptional regulators of MMP1 and the role of TET1 mutations in tumor progression. **A**, **B** KnockTF 2.0 database analysis identified TET1 as the most significant activator and KLF4 as the most significant repressor transcription factor of MMP1. **C** TET1 exhibited a high mutation frequency across various tumor types, with missense mutations being the most prevalent type. **D**–**G** Gene mutation and pathway analyses demonstrated a significant association between mutant TET1 and the EMT pathway in BRCA, COAD, LUAD, and KIRC.

Based on the KnockTF 2.0 database, TET1 was identified as the most significant activator transcription factor of MMP1, while KLF4 was recognized as the most significant repressor transcription factor (Fig. 5A, B). TET1 exhibited a notably high mutation frequency across various tumor types, with missense mutations being the most prevalent (Figs. 5C and S8). Gene mutation and pathway analyses revealed a significant association between mutant TET1 and the EMT pathway in BRCA, COAD, LUAD, and KIRC (Fig. 5D–G). Moreover, TET1 expression was elevated in some tumors and may be linked to tumor aggressiveness (Fig. S9). These findings suggested that TET1 instability in tumors may facilitate malignant cell escape, potentially through the activation of MMP1 transcription.

### Prognostic significance of MMP1 expression levels in pan-cancer analysis

**Fig. 6:**
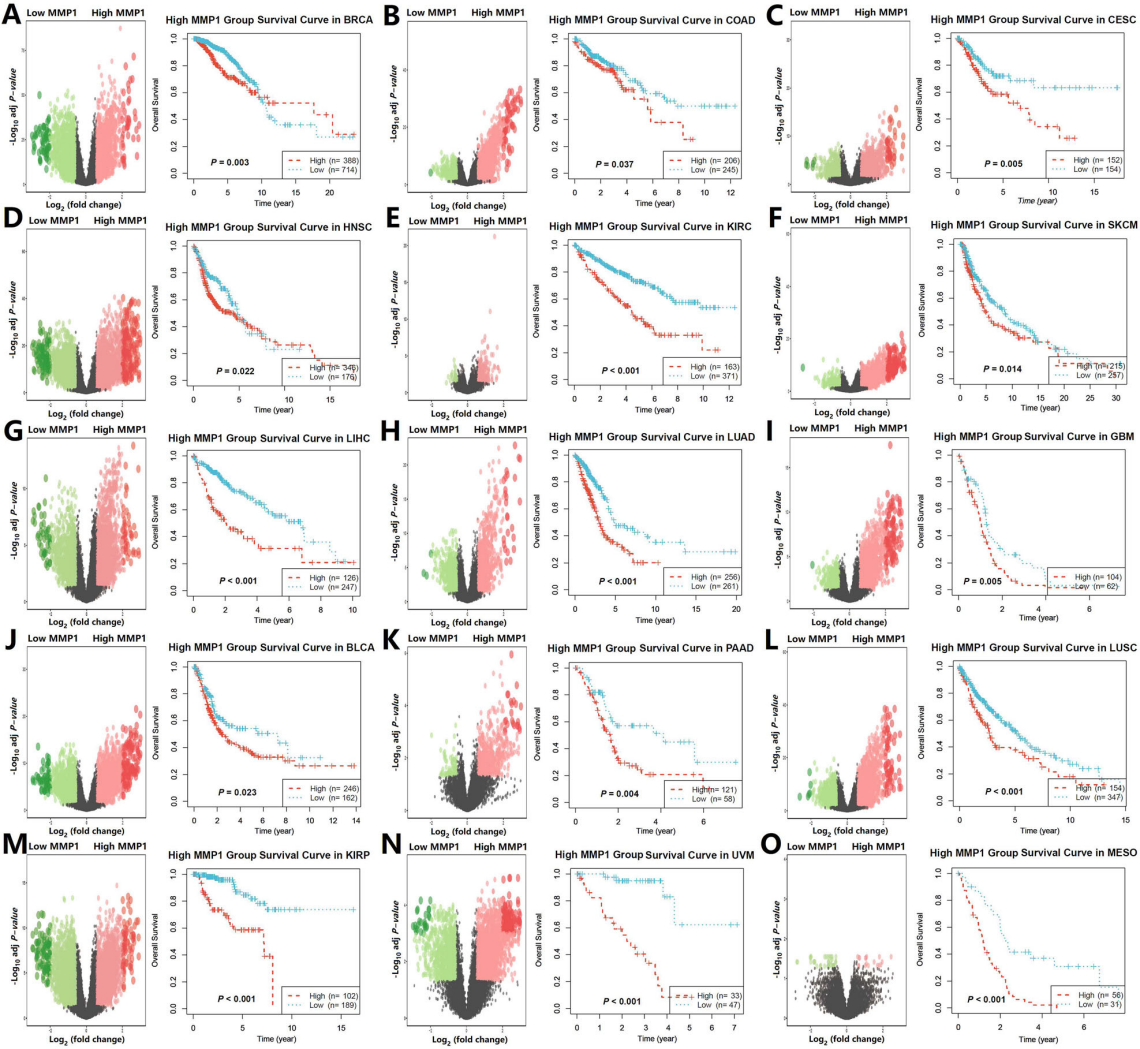
Expression differences and prognostic impact of MMP1 across pan-cancer. **A–O** Analysis of MMP1 expression across pan-cancer datasets revealed significant differences between high and low MMP1 expression groups. The gene set associated with high MMP1 expression was consistently linked to poor prognosis across multiple tumor types, emphasizing the critical role of MMP1 in tumor progression and its potential as a prognostic biomarker in cancer research.

Genes often function synergistically through co-activation with other genes. To further investigate the prognostic impact of MMP1 in tumors, we conducted a differential expression analysis comparing high and low MMP1 expression groups across pan-cancer datasets. The significantly upregulated genes in the high MMP1 expression group may contribute to the functional roles of MMP1. Notably, the gene set associated with high MMP1 expression was linked to poor prognosis across multiple tumor types (Fig. 6A–O).

### Therapeutic drug and drug sensitivity prediction based on MMP1

**Fig. 7:**
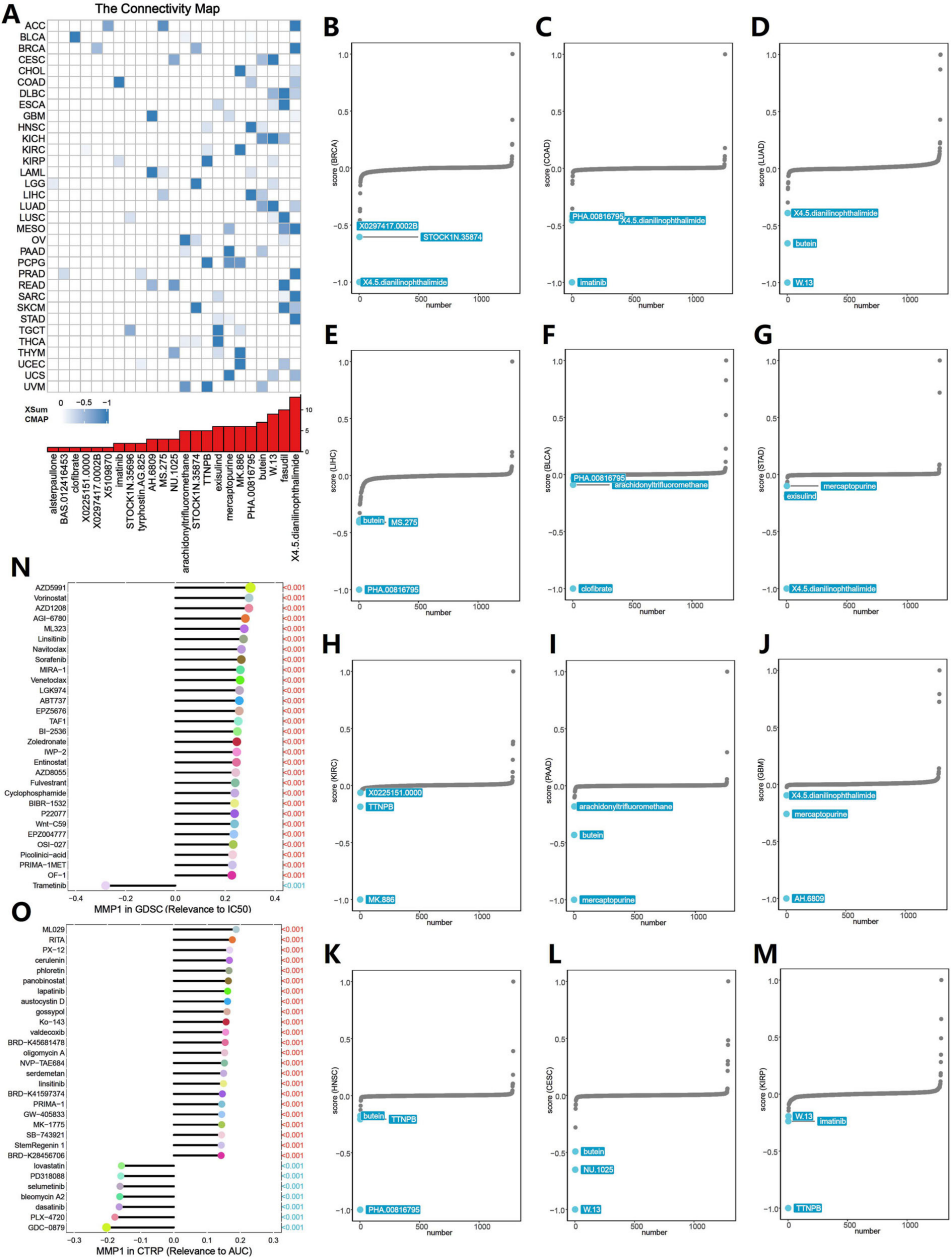
Identification of potential therapeutic drugs and drug resistance prediction based on MMP1 in pan-cancer. **A** Drug prediction was conducted using the CMap database and the XSum algorithm to identify small molecules capable of targeting the detrimental biological effects caused by dysregulated MMP1 expression. **B**–**M** Top four candidate drugs—X4.5.dianilinophthalimide, fasudil, W.13, and butein—highlighted as potential therapeutic agents for tumors with elevated MMP1 expression. **N** Drug Sensitivity analysis based on the GDSC database, demonstrating the association between high MMP1 expression and resistance to various therapeutic agents. **O** Confirmation of drug resistance patterns linked to MMP1 overexpression through the CTRP database, further validating the potential therapeutic challenges in high MMP1-expressing tumors.

In pan-cancer, based on the Connectivity Map (Cmap) database, we used the eXtreme Sum (XSum) algorithm to predict potential small molecules and drugs that could correct the detrimental biological effects caused by the dysregulated expression of MMP1 (Fig. 7A). The results suggested that X4.5.dianilinophthalimide, fasudil, W.13, and butein were the top four applicable drugs for treating tumors with high MMP1 expression in patients (Fig. 7B–M). The GDSC and CTRP databases indicated that elevated expression of MMP1 was associated with resistance to various drugs (Fig. 7N, O).

### Validation of MMP1 as a potential therapeutic target in tumors

**Fig. 8:**
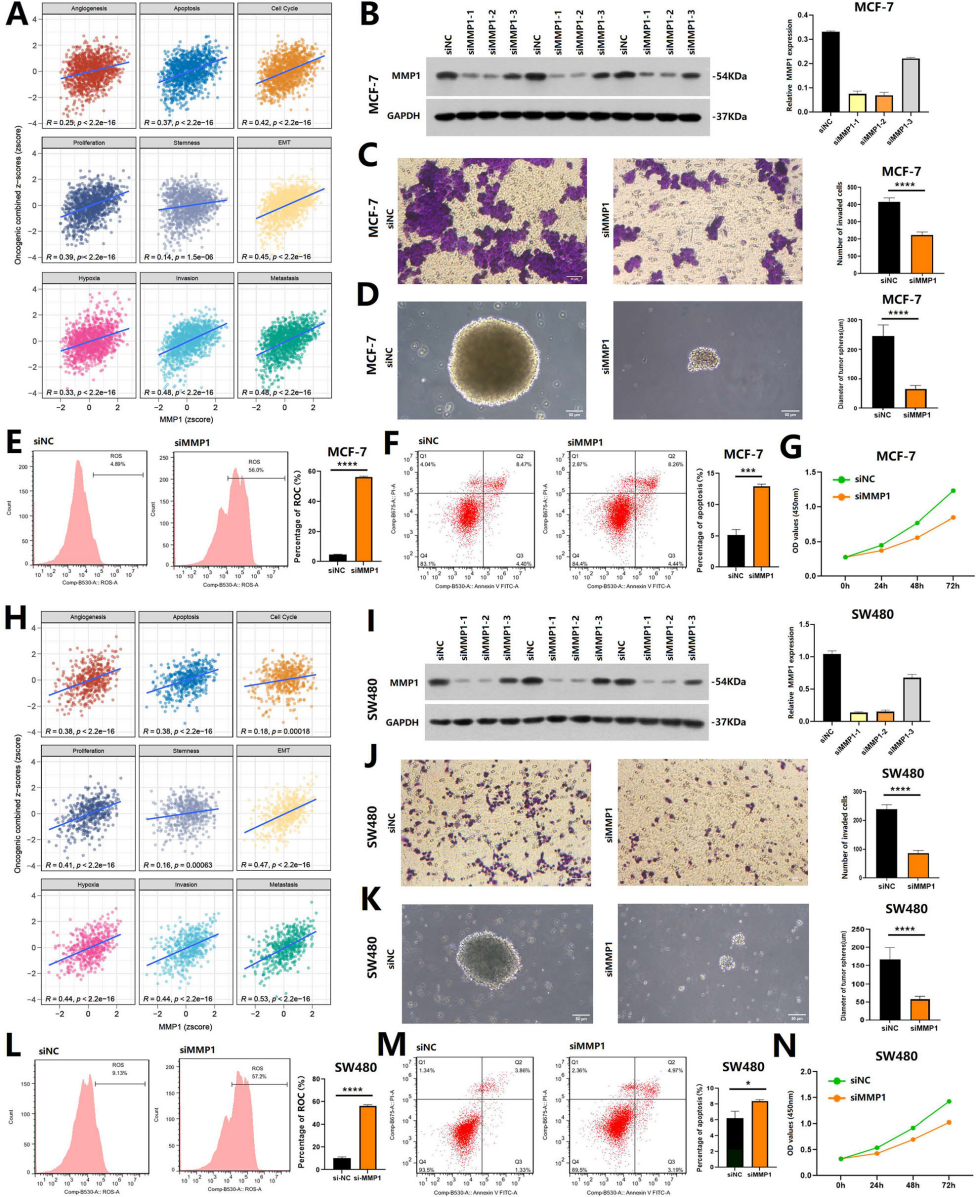
The Role of MMP1 in mediating malignant phenotypes in BRCA and COAD. **A**, **H** Tumor status analysis using the CancerSEA database revealed that MMP1 plays a critical role in mediating various malignant phenotypes in BRCA and COAD, including cell apoptosis, proliferation, stemness, and invasion. **B**, **I** Western blot analysis of MCF-7 and SW480 cell lines identified the optimal protein bands following MMP1 knockdown. **C**–**G**, **J**–**N** Functional assays comparing the siMMP1 group to the siNC group demonstrated significantly reduced cell invasion capability and impaired stemness in the siMMP1 group. Knockdown of MMP1 also resulted in increased ROS levels, elevated apoptosis, and a decreased cell proliferation rate.

Tumor status analysis using the CancerSEA database revealed that MMP1 played a critical role in mediating various malignant phenotypes in BRCA and COAD, including cell apoptosis, proliferation, stemness, and invasion (Fig. 8A, H). To further investigate, we conducted a series of MMP1 knockdown assays in tumor cell lines (MCF-7 and SW480). WB analysis was performed to identify the optimal protein bands (Fig. 8B, I, Original Data). Compared to the siNC group, the siMMP1 group exhibited significantly reduced cell invasion capability, impaired stemness, increased levels of ROS, elevated cell apoptosis, and a decreased cell proliferation rate (Fig. 8C–G, J–N). These findings suggested that, beyond its well-known role in EMT, MMP1 was a key driver of multiple malignant behaviors, further establishing its potential as a powerful therapeutic target for aggressive tumors.

## Discussion

The abnormal expression of MMP1 is a hallmark feature in many malignant tumors. While much of the existing research has centered on MMP1’s role in facilitating tumor cell invasion through the stroma and blood vessel walls to drive metastasis [12], its critical involvement in tumor immunology warrants greater attention. Through large-scale ST and scRNA-seq analyses, we identified that elevated MMP1 expression in malignant cells not only enhances their EMT capabilities but also facilitates significant interactions between tumor cells and immune cells. We also found that MMP1 mediated the activation of other signaling pathways in tumors, such as the KRAS signaling pathway, the inflammatory response signaling pathway, and the hypoxia pathway, suggesting its mediating ability in other malignant behaviors, such as tumor initiation and survival, besides mediating metastasis. These interactions are closely linked to phenotypic alterations in tumor-associated macrophages (TAMs) and the functional impairment of CD8+ T cells. Together, these changes create a tumor-promoting microenvironment that fosters immune evasion and tumor progression. This dual role of MMP1 in driving both structural and immune changes highlights its importance as a key modulator of the TME and underscores its potential as a therapeutic target.

Yu et al. [13] reported that macrophage-mediated upregulation of MMP1 expression drived the proliferation of colon tumor cells. Similarly, previous studies have shown that macrophages can regulate inflammatory-stromal interactions in the TME primarily via activation of the TNFα/NF-κB signaling pathways [14]. Consistent with these findings, our analysis revealed significant activation of the TNFα/NF-κB signaling pathways in pan-cancer samples with high MMP1 expression, which was positively correlated with MMP1 overexpression. Further pan-cancer ST and scRNA-seq data demonstrated that MMP1 expression was predominantly localized in malignant cells. Notably, MMP1+ malignant cells exhibited stronger outgoing signals to macrophages, particularly through the ANXA1-FPR3 signaling axis. ANXA1 interacts with various cell types within the TME, influencing tumor proliferation, invasion, and metastasis [15, 16]. Previous research has also shown that malignant cells polarize macrophages through the ANXA1-FPR3 signaling axis, and these polarized macrophages, in turn, enhance tumor invasion and metastatic potential [17]. Additionally, this axis plays a key role in driving macrophage polarization towards the M2 phenotype, which is characterized by immunosuppressive functions [18, 19]. M2 macrophages promote tumor progression by secreting anti-inflammatory cytokines like TGF-β, contributing to immune suppression and facilitating tissue remodeling within the TME [20]. Therefore, we proposed a bidirectional regulatory loop in the microenvironment, where MMP1 not only responded to NF-κB and EMT pathways but also fed back into these pathways, amplifying tumor-promoting effects. MMP1, a downstream effector, was upregulated by both EMT and TNFα/NF-κB signaling. EMT induced changes in cellular adhesion and promoted a migratory phenotype, leading to MMP1 upregulation [21]. Similarly, TNFα and NF-κB activation also induced MMP1 expression [22]. Notably, MMP1 activated macrophages via the ANXA1-FPR3 pathway, triggering TNFα secretion, which further enhanced NF-κB activation and tumor progression. MMP1-driven matrix degradation also released TNFα [23], creating a feedback loop that sustained MMP1 expression. This complex interaction highlighted the potential of targeting these feedback loops for therapeutic intervention in tumor.

The upregulation of MMP1 was strongly associated with impaired T-cell function and the induction of immunosuppression [24, 25]. MMP1+ malignant cells exhibited enhanced efferent signaling to CD8+ T cells, particularly through activation of the CXCL16-CXCR6 signaling axis. This observation highlighted a critical link between MMP1’s role in tumor biology and its influence on immune cell trafficking and functionality. The CXCL16-CXCR6 axis displayed dual characteristics within the TME. While it can promote T cell-mediated anti-tumor immune responses, it also contributes to T-cell dysfunction, thereby facilitating immunosuppression [26–28]. CXCR6, a chemokine receptor, primarily directs the migration of CD8+ T cells to specific microenvironments through its interaction with its ligand, CXCL16 [29]. In the TME or during chronic infections, CXCL16 was often highly expressed in tumor tissues, infected areas, or sites of inflammation [30, 31]. This interaction recruited CD8+ T cells to these microenvironments via CXCR6 binding to CXCL16. However, we speculated that continuous antigen exposure coupled with immunosuppressive signaling within these localized environments may drive excessive immune suppression, resulting in the depletion and dysfunction of CD8+ T cells. Consequently, CXCR6 played a pivotal role in CD8+ T-cell dysfunction by anchoring them in specific immunosuppressive niches within the TME, contributing to immune evasion by tumors. It was noteworthy that we also observed a close correlation between MMP1 expression and neutrophil infiltration in the tumor microenvironment in certain cancer types. Neutrophils promoted the spread and migration of tumor cells by secreting matrix metalloproteinases, which degraded the ECM surrounding the tumor tissue [32, 33]. The activation of neutrophils and the secretion of MMP1 played a significant role in tumor-immune evasion and tumor progression. Future work could explore these additional immune cell types to gain a deeper understanding of how MMP1 modulates the entire TME.

## Conclusion

The presence of MMP1+ malignant cells in the TME suggested that MMP1 not only facilitated tumor cell invasion and metastasis, but also played a critical role in immune evasion by modulating the function of immune cells, including macrophages and CD8+ T cells. Targeting MMP1 and its downstream signaling pathways, such as the ANXA1-FPR3 and CXCL16-CXCR6 axes, represented a promising therapeutic strategy to disrupt these tumor-promoting mechanisms and enhance anti-tumor immunity.

## Materials and methods

### Data source

The ST data, encompassing 11 types of tumors from 11 patients, were obtained from 10X Genomics platform, along with their corresponding accession IDs. Additionally, the scRNA-seq data, including 42 types of tumors from 118 scRNA-seq datasets, were downloaded from TISCH2 database, complete with accession IDs (Table S1). Survival information and transcriptomic expression data for various types of tumors were obtained from the TCGA database.

### Data processing

Data processing of ST: We employed deconvolution analysis techniques to assess the cellular composition [34]. Based on the results obtained from this deconvolution, we identified the predominant cell type in each microregion. The SpatialDimPlot function from the Seurat package was used to visualize the dominant cellular composition for each microregion. Additionally, the SpatialFeaturePlot function from the Seurat package enabled us to visualize the gene expression landscape within each microregion. We performed Spearman correlation analysis to evaluate the relationships between cellular compositions across all spots, as well as the correlations between cellular compositions and gene expression levels, utilizing the linkET package for visualization.

Data processing of scRNA-seq data: Gene expression data were acquired at the pan-cancer single-cell resolution from the TISCH2 database [35]. We employed the pheatmap package to generate a heatmap that visualized the gene expression landscape across various tumor single-cell types. Batch effects were corrected using the Harmony [36]. The UMAP technique was applied to visualize the classification results for the single-cell subsets. Cellchat software was used to analyze the cell-cell communication [37].

### Tumor status assessment

Based on the CancerSEA database, we organized the different functional statuses of 14 tumor cell types [38]. Using the z-score parameter from the GSVA R package, we calculated the gene sets for these 14 functional statuses and obtained the combined z-score values [39]. Subsequently, we further standardized these scores using the scale function, defining them as gene set scores. Finally, we calculated the Pearson correlation between MMP1 and each gene set score.

### Analysis of MMP1 expression and prognosis in tumors

We paired the TPM expression levels from normal samples in GTEx with those from tumor samples in TCGA. Using the gganatogram package, we visualized the median z-scores of various organs for both tumor and normal groups. We extracted tumor data from TCGA and used a cutoff value of 0.3 to divide the samples into two groups. Differential analysis was conducted using the limma package, and Kaplan–Meier survival analysis was performed to compare the high and low MMP1 expression groups (fold change > 1) utilizing the survival package.

### Analysis of MMP1 immunological significance, metabolic, and hallmark characteristics in tumors

Based on the quartiles of MMP1 expression, all patients were divided into four types. Q1 represented the samples in the highest 25% of specific gene expression levels, while Q4 represented the samples in the lowest 25% of expression levels. Following the research by Thorsson V et al. on immune responses and genomic status, the average score for each type of patient was calculated [40]. The Spearman correlation analysis was used to calculate the correlation between MMP1 expression and tracking tumor immunophenotype (TIP) scores, as well as the autocorrelation between TIP scores [41]. The linkET package was used for visualization. The data for immune infiltration analysis was collected from TIMER2.0, which demonstrated the relationship between different cell types and MMP1 expression under various algorithms [42]. The cor.test function was used to perform Pearson correlation analysis between each immune gene and MMP1. The GSEA function from the clusterProfiler package was employed to conduct gene set enrichment analysis on the Hallmark gene set and the KEGG metabolic gene set, using the high and low expression groups of MMP1 [43].

### Inference of key signaling pathways

PROGENy software was used to assess the activity differences of TNFα and NF-κB signaling pathways between high and low MMP1 expression groups. The cor.test function was used to compute the Pearson correlation coefficient between two genes. Simultaneously, we conducted Fisher’s exact test to further validate the correlation. Furthermore, we calculated the correlation between MMP1 expression and the expression of key EMT markers (SNAL1 and VIM) based on the quartiles of MMP1 expression. The Genomic Identification of Significant Targets in Cancer, version 2 (GISTIC2) score was used to determine copy number changes in key genes associated with the pathway.

### Transcriptional regulation and mutation analysis

KnockTF 2.0 was a database that collected expression profiles following the knockdown of transcription factors [44]. We utilized the KnockTF 2.0 database to predict the impact of transcription factor changes on MMP1 expression. We gathered single-nucleotide variant (SNV) data from 33 cancer types available in TCGA and calculated the mutation frequency of SNVs within the coding regions of MMP1, TET1, and KLF4. Using the maftools package, we generated oncoplot diagrams to visualize these mutations. Based on Cbioportal database, gene mutation sites and mutation frequency differences were mapped. Patients were categorized into mutation and wild-type groups based on the presence of mutations in TET1. Differential analysis was conducted using the limma package, while gene set enrichment analysis was performed using the clusterProfiler package, focusing on the Hallmark gene set and KEGG metabolic gene set.

### Drug sensitivity and therapeutic drug screening

We utilized the Cmap database and employed the XSum to compare MMP1-related features with Cmap gene features [45, 46]. This comparison yielded similarity scores for 1,288 compounds. Compounds with lower similarity scores may potentially inhibit gene-mediated oncogenic effects. We performed a Spearman correlation analysis to assess the relationship between MMP1 expression levels and the AUC values in the CTRP database. Additionally, we evaluated the correlation between MMP1 expression in the GDSC database and the IC50 values of the measured antagonists.

### Vulnerability validation of targeting MMP1

The cell lines (MCF-7 and SW480 cells) sourced from the American Type Culture Collection have been validated by STR profiling and confirmed to be free of mycoplasma contamination. The MMP1 transient knockdown in MCF-7 and SW480 cells was performed using the siRNA kit (Huzhou Hippo Biotechnology Co., Ltd., China), and the knockdown efficiency was verified by western blot (WB). The primary antibodies used for WB were MMP1 antibody (Abcam, Cambridge, UK) and GAPDH antibody (Shanghai KangChen Biotechnology Co., Ltd., China). The final effective sequence identified was: siMMP1(hMMP1 si-1 sense: CUGAACAGCCCAGUACUUATT and hMMP1 si-1 antisense: UAAGUACUGGGCUGUUCAGTT). The cell invasion assay of MCF-7 and SW480 cells in the siNC group and the siMMP1 group primarily involved adding diluted Matrigel (Corning Inc., USA) to the invasion chamber to simulate the ECM, and crystal violet (Amresco Inc., USA) staining was used to visualize cell invasion behavior.

The cell spheroid formation assay involved adding a mixture of Lipo2000 (Thermo Fisher, USA) and RNA to the cells, followed by seeding the cells onto a non-adherent plate to observe spheroid formation. The cell reactive oxygen species (ROS) assay uses the fluorescent properties of the DCFH-DA kit (Beyotime, China) to detect ROS levels in MCF-7 and SW480 cells. The cell apoptosis detection was achieved through the Annexin V-FITC and Propidium Iodide (PI) double staining method, followed by analysis using flow cytometry. The cell proliferation assay was detected by the Cell Counting Kit-8 (CCK-8) method. In the CCK-8 assay, cell proliferation was assessed at the indicated time points (0 h, 24 h, 48 h, and 72 h) by adding CCK-8 reagent (Suzhou Youyi Landi Biotechnology Co., Ltd., China) and measuring the absorbance.

### Statistical analysis

In this study, the Student’s t-test was employed to analyze data from cell functional experiments. The data met the assumption of similar variance, and all experiments were conducted in triplicate. The Wilcoxon rank-sum test was employed to compare two groups in bioinformatics analysis. The thresholds for significance were established at *P < 0.05, **P < 0.01, ***P < 0.001, and ****P < 0.0001. Analyses were conducted using R software, version 4.3.3.

## Supplementary information

Fig. S1

Fig. S2

Fig. S3

Fig. S4

Fig. S5

Fig. S6

Fig. S7

Fig. S8

Fig. S9

Original Data

Table S1

## Data Availability

The data that support the findings of this study are available from the corresponding author upon reasonable request.
