# Supplementary material for: Decoding the impact of MMP1+ malignant subsets on tumor-immune interactions: insights from single-cell and spatial transcriptomics
Source: Cell Death Discov. 2025 May 20;11:244. doi: 10.1038/s41420-025-02503-y (PMC12092693; doi:10.1038/s41420-025-02503-y)
Supplement: Supplementary file 2 — Fig. S2 [file 41420_2025_2503_MOESM2_ESM.pdf]

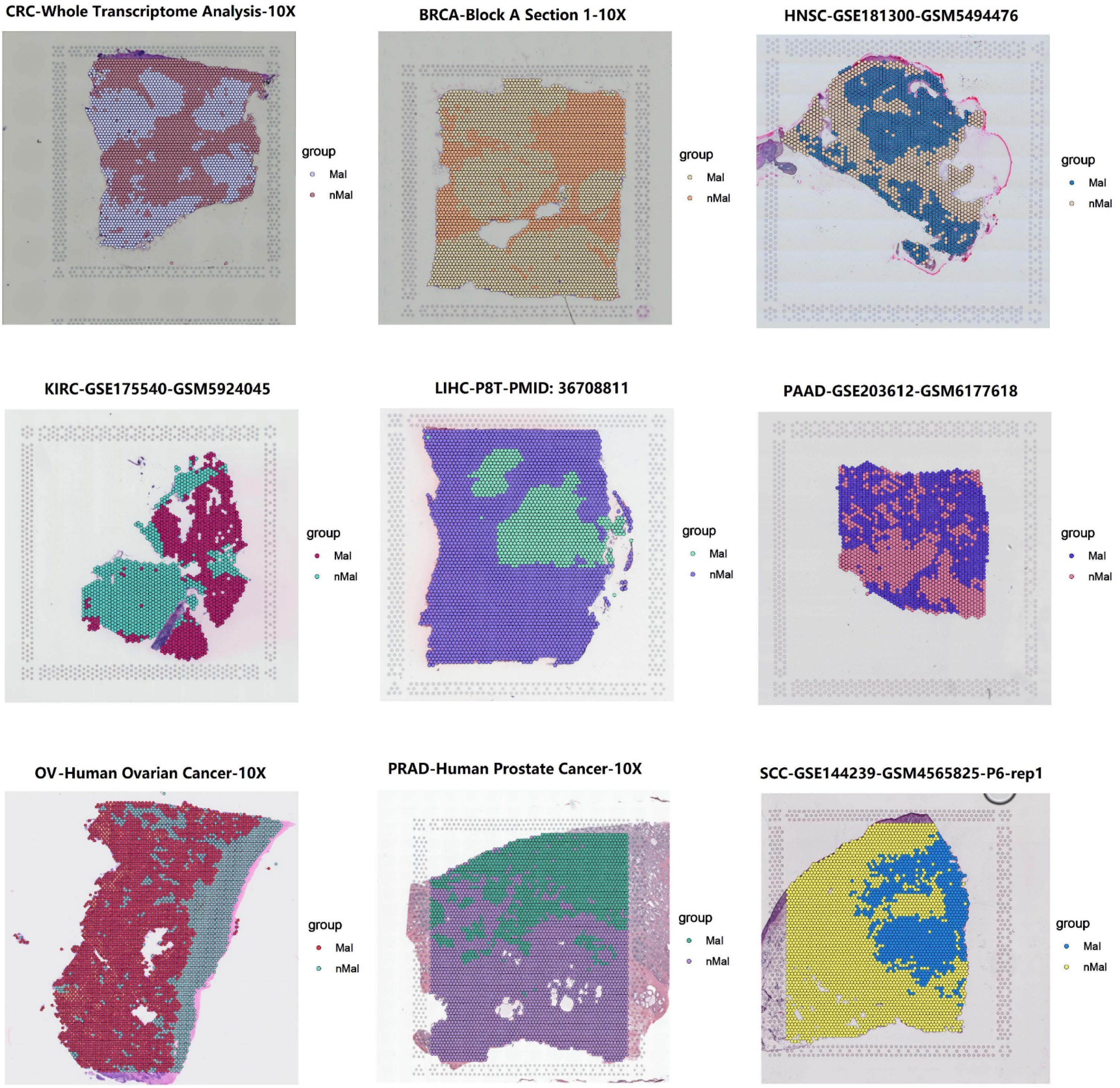

Fig. S2. Classification of tissue regions into malignant (Mal) and non-malignant (nMal) based on the proportion of malignant cells in each microregion
