# Supplementary figures and images for: Decoding the impact of MMP1+ malignant subsets on tumor-immune interactions: insights from single-cell and spatial transcriptomics
Source: Cell Death Discov. 2025 May 20;11:244. doi: 10.1038/s41420-025-02503-y (PMC12092693; doi:10.1038/s41420-025-02503-y)

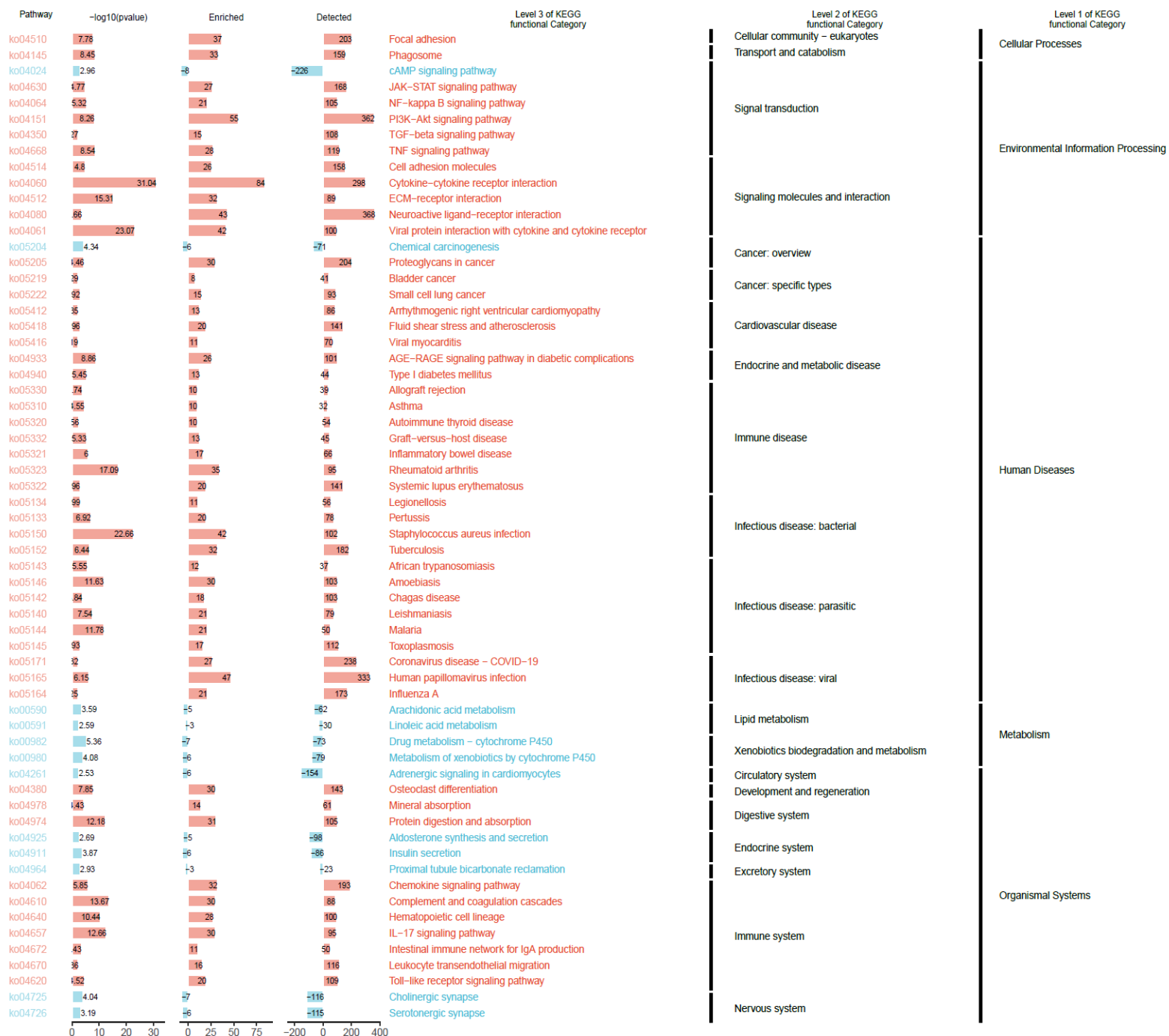

**Fig. S3. KEGG pathway enrichment difference of high and low MMP1 expression groups**

Supplement: Supplementary file 3 — Fig. S3 [file 41420_2025_2503_MOESM3_ESM.pdf]

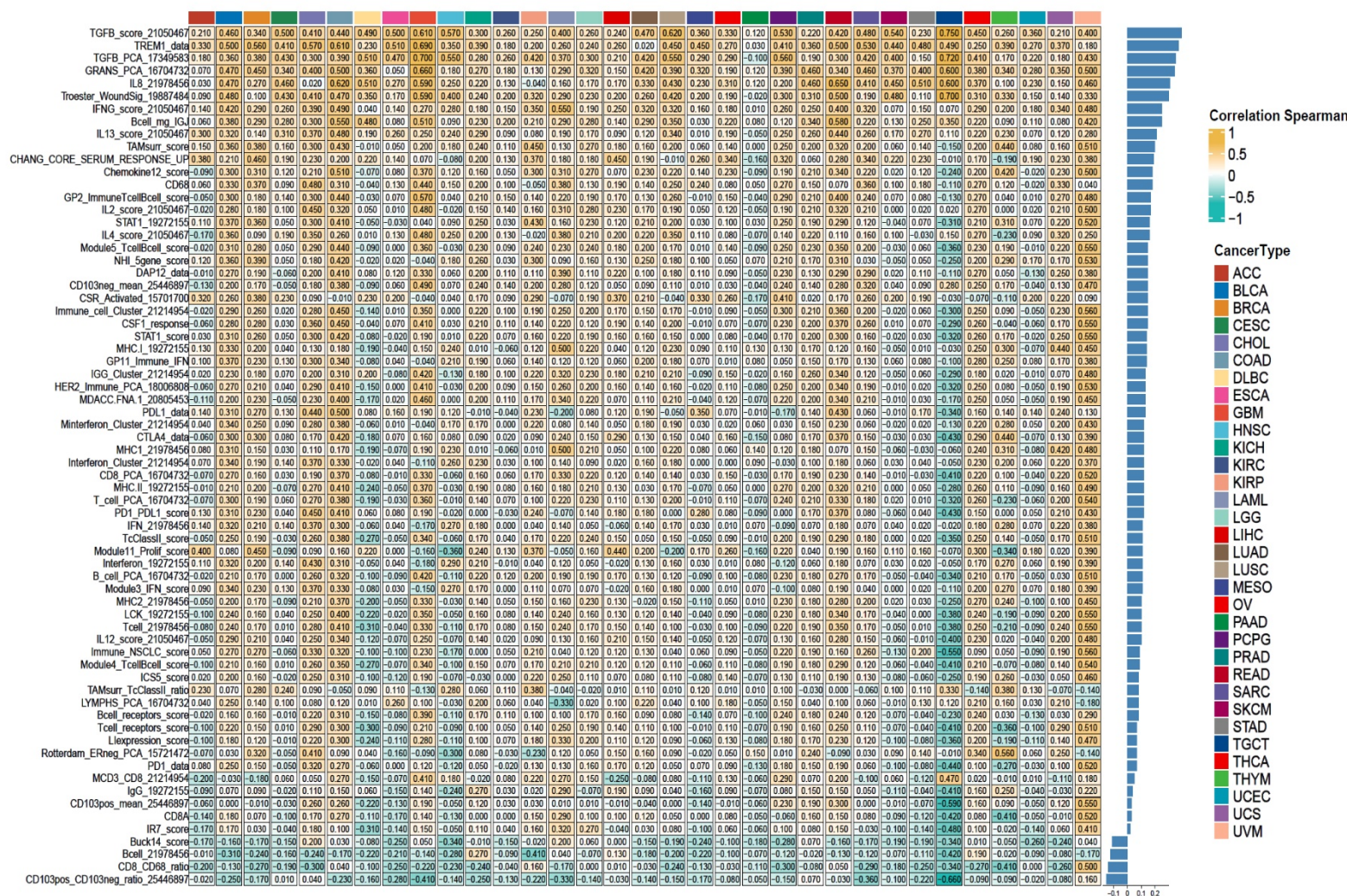

Fig. S5. Heatmap of the correlation between the MMP1 gene and immune signatures

Supplement: Supplementary file 5 — Fig. S5 [file 41420_2025_2503_MOESM5_ESM.pdf]

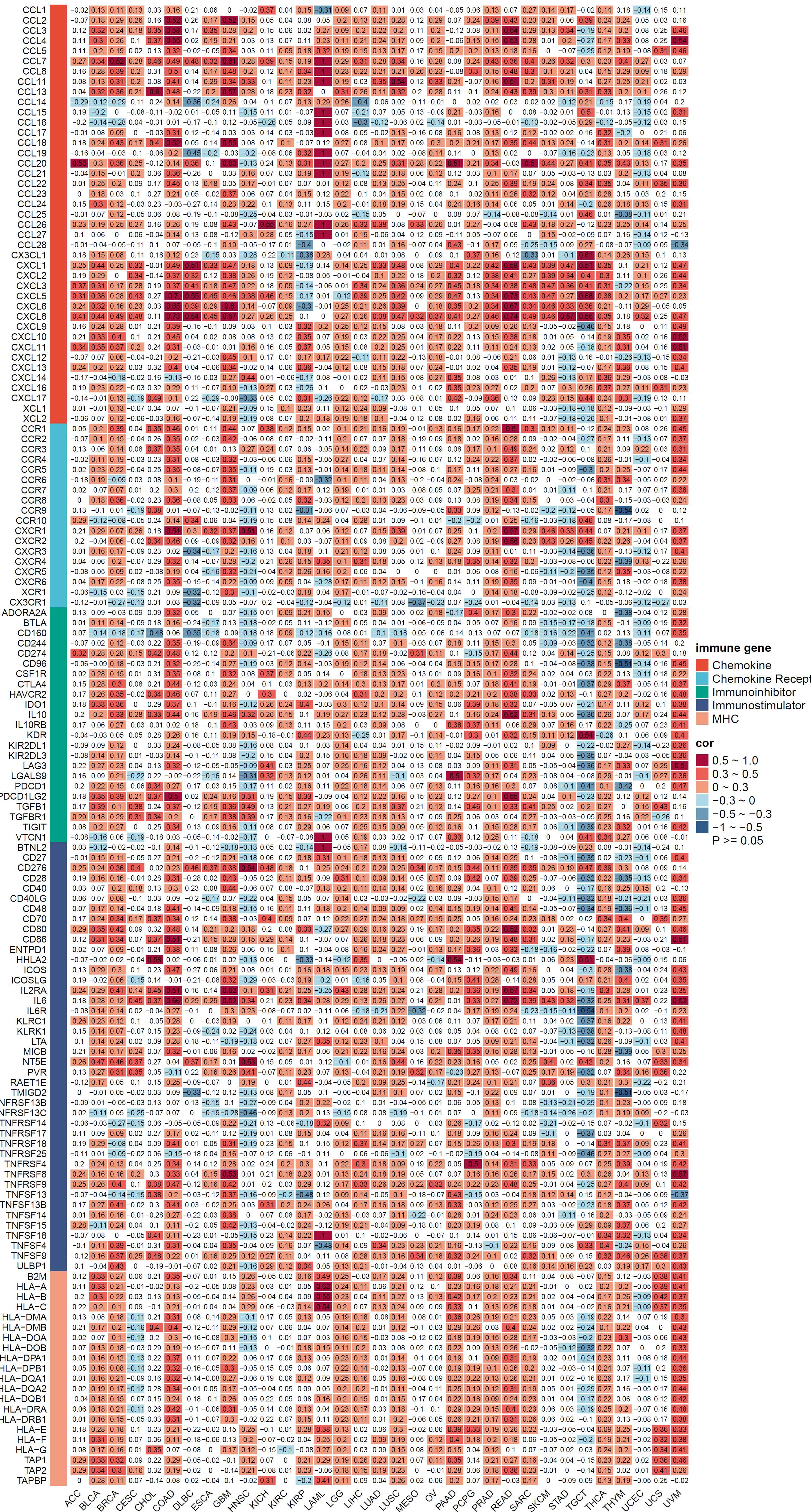

Supplement: Supplementary file 6 — Fig. S6 [file 41420_2025_2503_MOESM6_ESM.pdf]

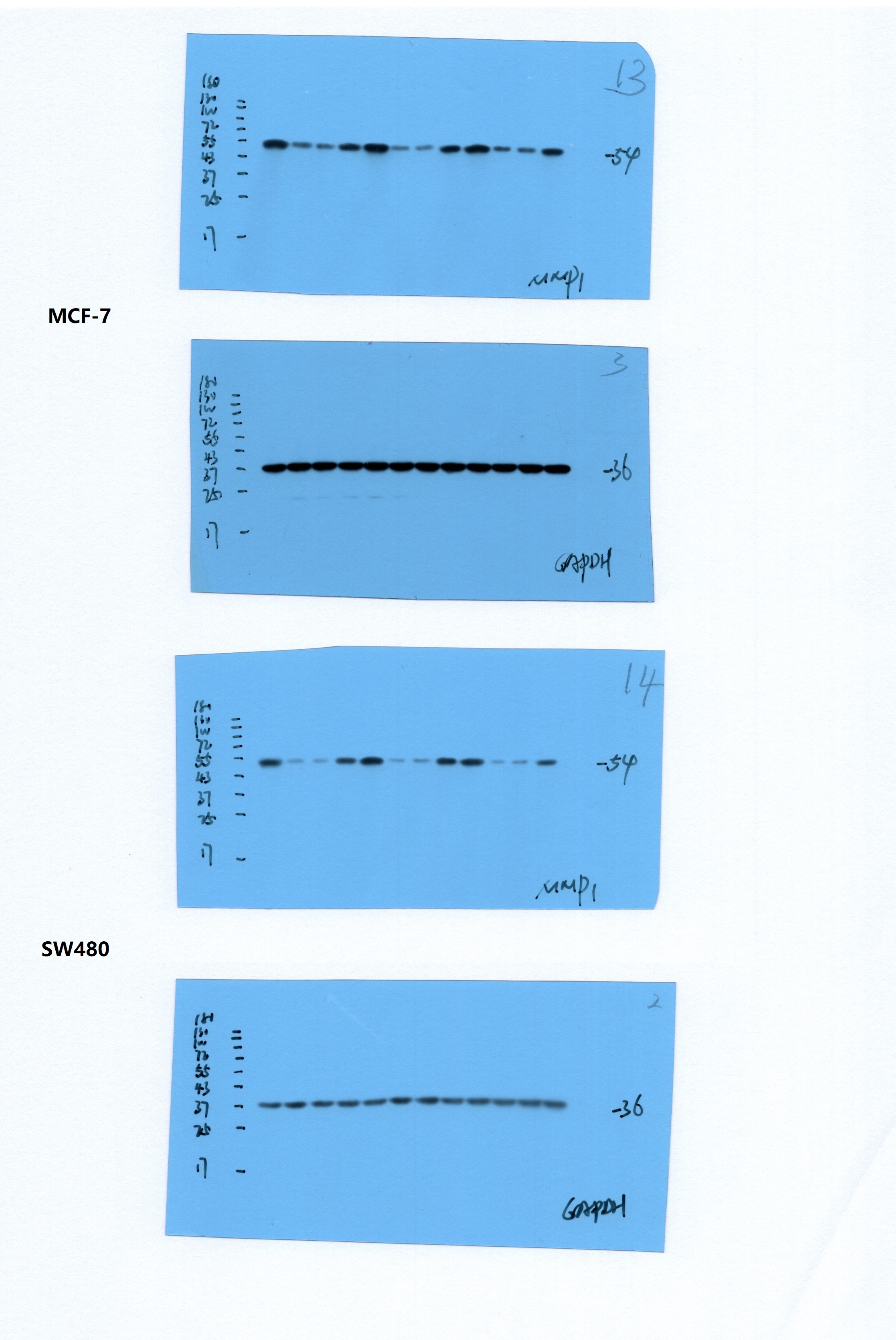

Supplement: Supplementary file 10 — Original Data [file 41420_2025_2503_MOESM10_ESM.jpg]
