# Supplementary material for: Decoding the impact of MMP1+ malignant subsets on tumor-immune interactions: insights from single-cell and spatial transcriptomics
Source: Cell Death Discov. 2025 May 20;11:244. doi: 10.1038/s41420-025-02503-y (PMC12092693; doi:10.1038/s41420-025-02503-y)
Supplement: Supplementary file 4 — Fig. S4 [file 41420_2025_2503_MOESM4_ESM.pdf]

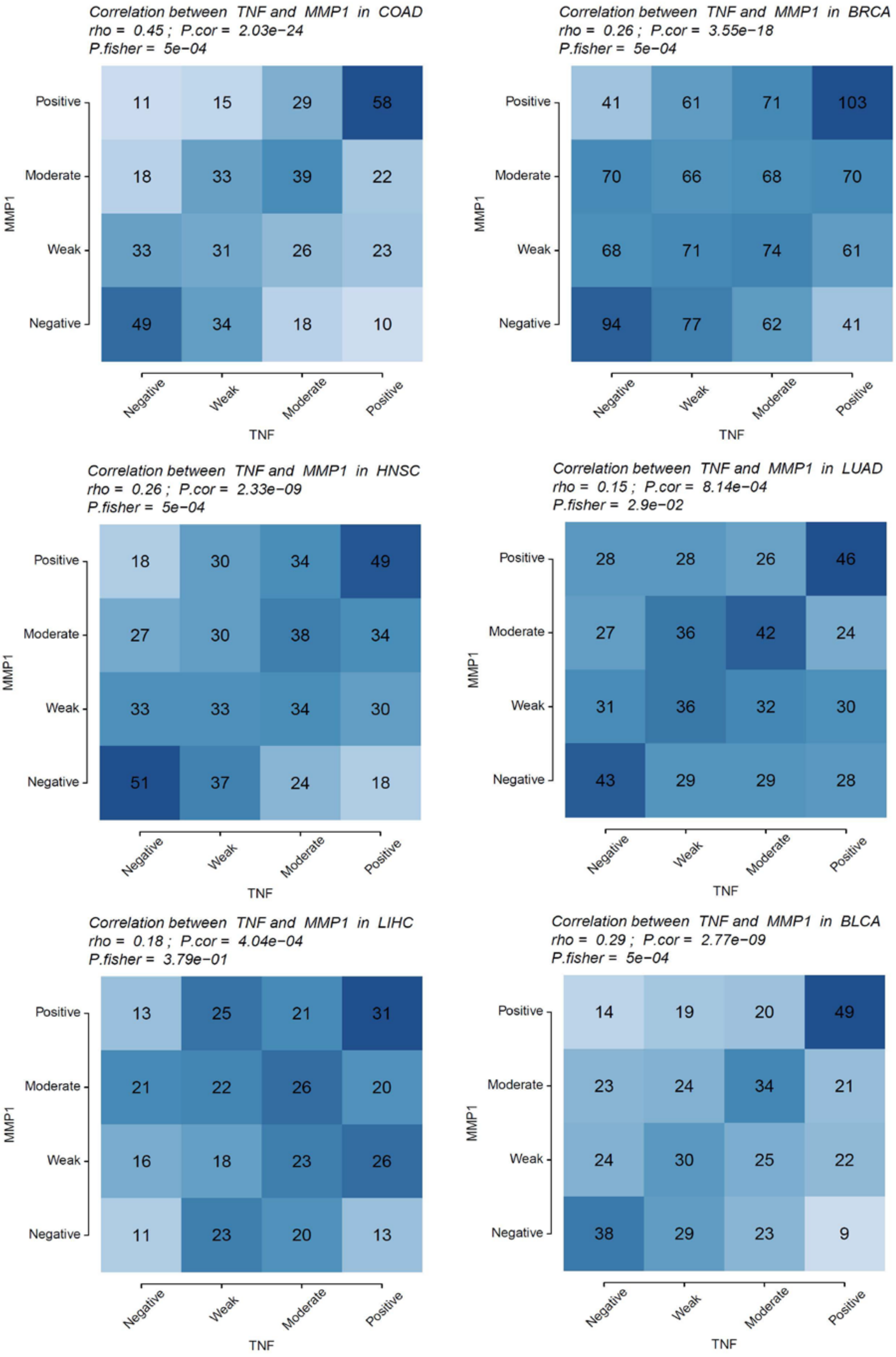

Fig. S4. Correlation analysis of MMP1 and TNF expression with Fisher's exact test across different tumors
