# Supplementary material for: Decoding the impact of MMP1+ malignant subsets on tumor-immune interactions: insights from single-cell and spatial transcriptomics
Source: Cell Death Discov. 2025 May 20;11:244. doi: 10.1038/s41420-025-02503-y (PMC12092693; doi:10.1038/s41420-025-02503-y)
Supplement: Supplementary file 7 — Fig. S7 [file 41420_2025_2503_MOESM7_ESM.pdf]

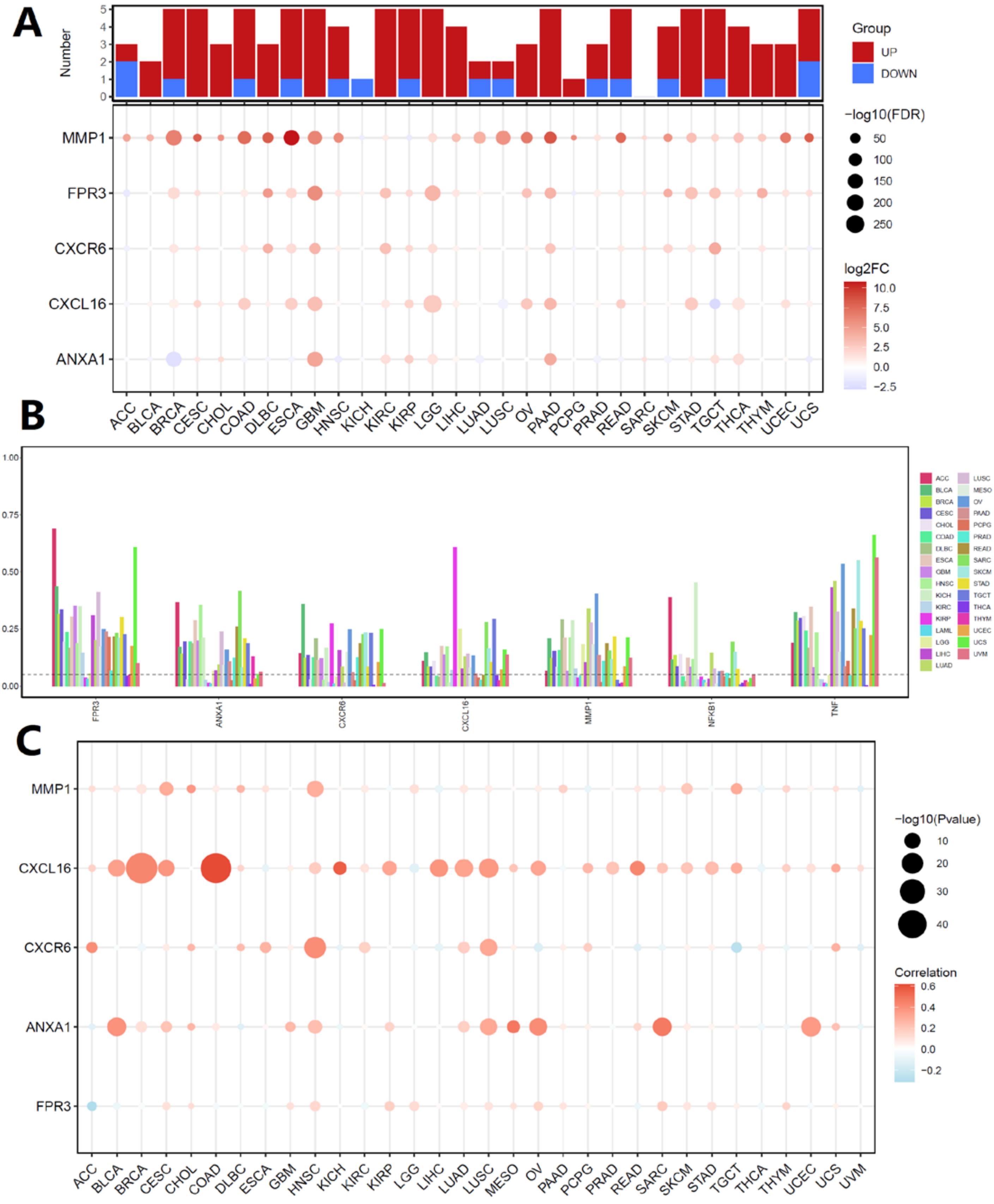

Fig. S7. CXCL16-CXCR6 and ANXA1-FPR3 pathways as key mechanisms across tumor types

(A) The expression levels of CXCL16-CXCR6 and ANXA1-FPR3 signaling pathway components across various tumor types.

(B) The copy number variation of CXCL16-CXCR6 and ANXA1-FPR3 signaling pathway components.

(C) The expression correlation of CXCL16-CXCR6 and ANXA1-FPR3 signaling pathway components across various tumor types.
