# Supplementary material for: Decoding the impact of MMP1+ malignant subsets on tumor-immune interactions: insights from single-cell and spatial transcriptomics
Source: Cell Death Discov. 2025 May 20;11:244. doi: 10.1038/s41420-025-02503-y (PMC12092693; doi:10.1038/s41420-025-02503-y)
Supplement: Supplementary file 8 — Fig. S8 [file 41420_2025_2503_MOESM8_ESM.pdf]

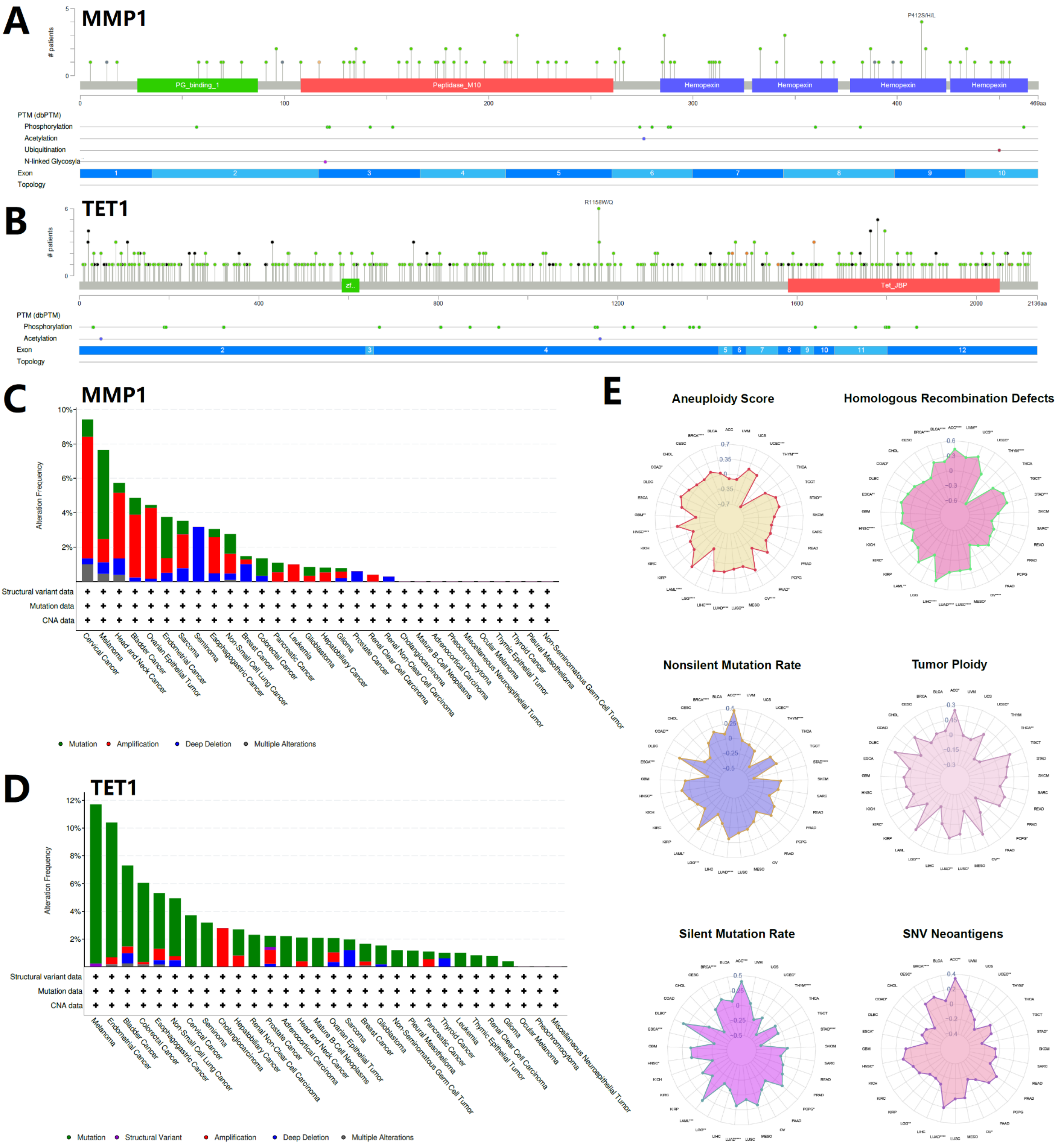

Fig. S8. Analysis of MMP1 and TET1 mutations in pan-cancer

(A, B) Mutation sites of MMP1 and TET1.

(C, D) Distribution of MMP1 and TET1 mutations across various cancers.

(E) Correlation analysis of TET1 expression and different types of genomic variation across various tumors.
