# Supplementary material for: Decoding the impact of MMP1+ malignant subsets on tumor-immune interactions: insights from single-cell and spatial transcriptomics
Source: Cell Death Discov. 2025 May 20;11:244. doi: 10.1038/s41420-025-02503-y (PMC12092693; doi:10.1038/s41420-025-02503-y)
Supplement: Supplementary file 9 — Fig. S9 [file 41420_2025_2503_MOESM9_ESM.pdf]

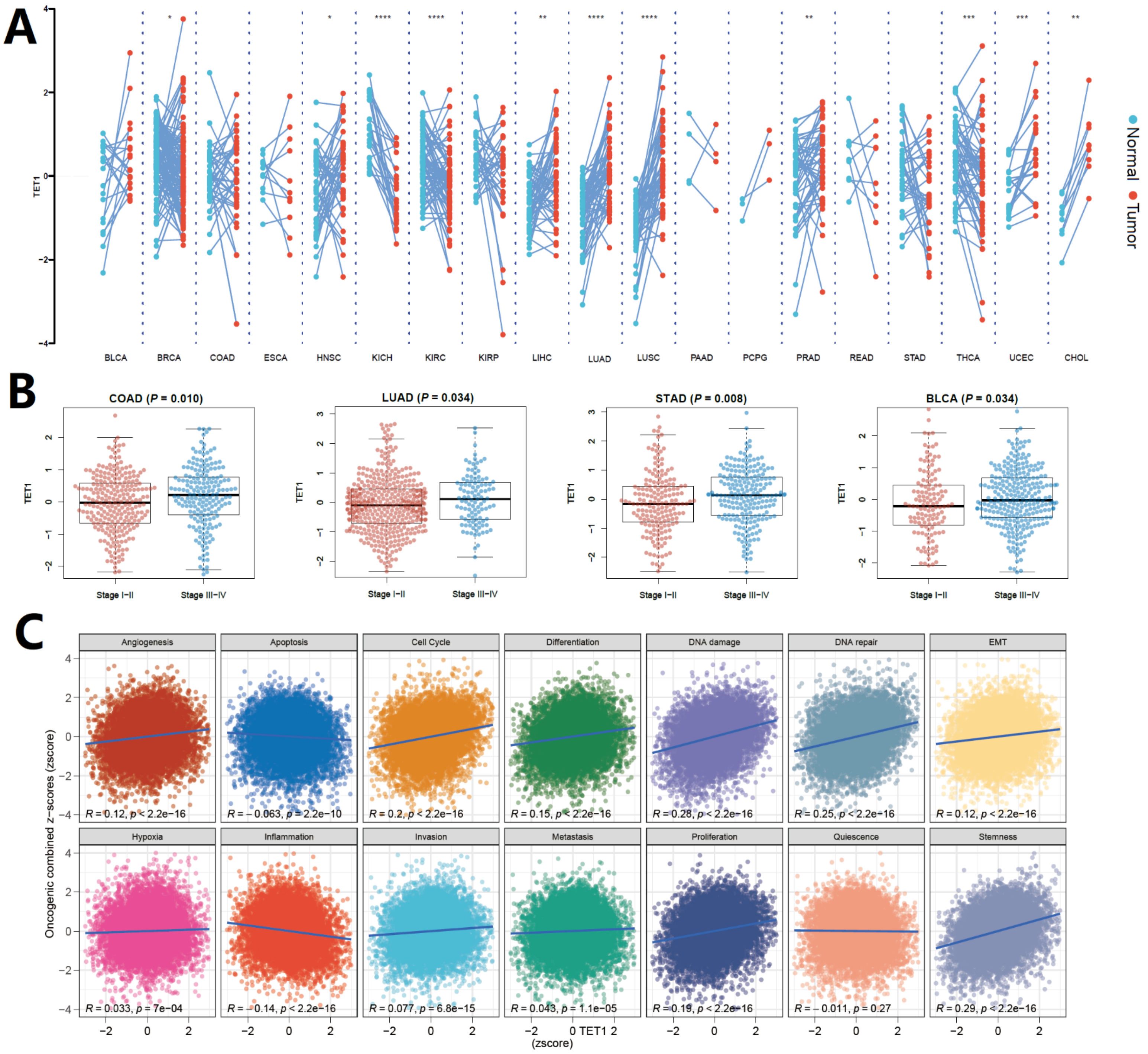

Fig. S9. Pan-cancer analysis of TET1 signature

(A) The expression of TET1 in pan-cancer based on paired samples from TCGA.

(B) The expression of TET1 at different tumor stages.

(C) The correlation between TET1 expression and malignant features of tumors.
