# Supplementary material for: Decoding the impact of MMP1+ malignant subsets on tumor-immune interactions: insights from single-cell and spatial transcriptomics
Source: Cell Death Discov. 2025 May 20;11:244. doi: 10.1038/s41420-025-02503-y (PMC12092693; doi:10.1038/s41420-025-02503-y)
Supplement: Supplementary file 1 — Fig. S1 [file 41420_2025_2503_MOESM1_ESM.pdf]

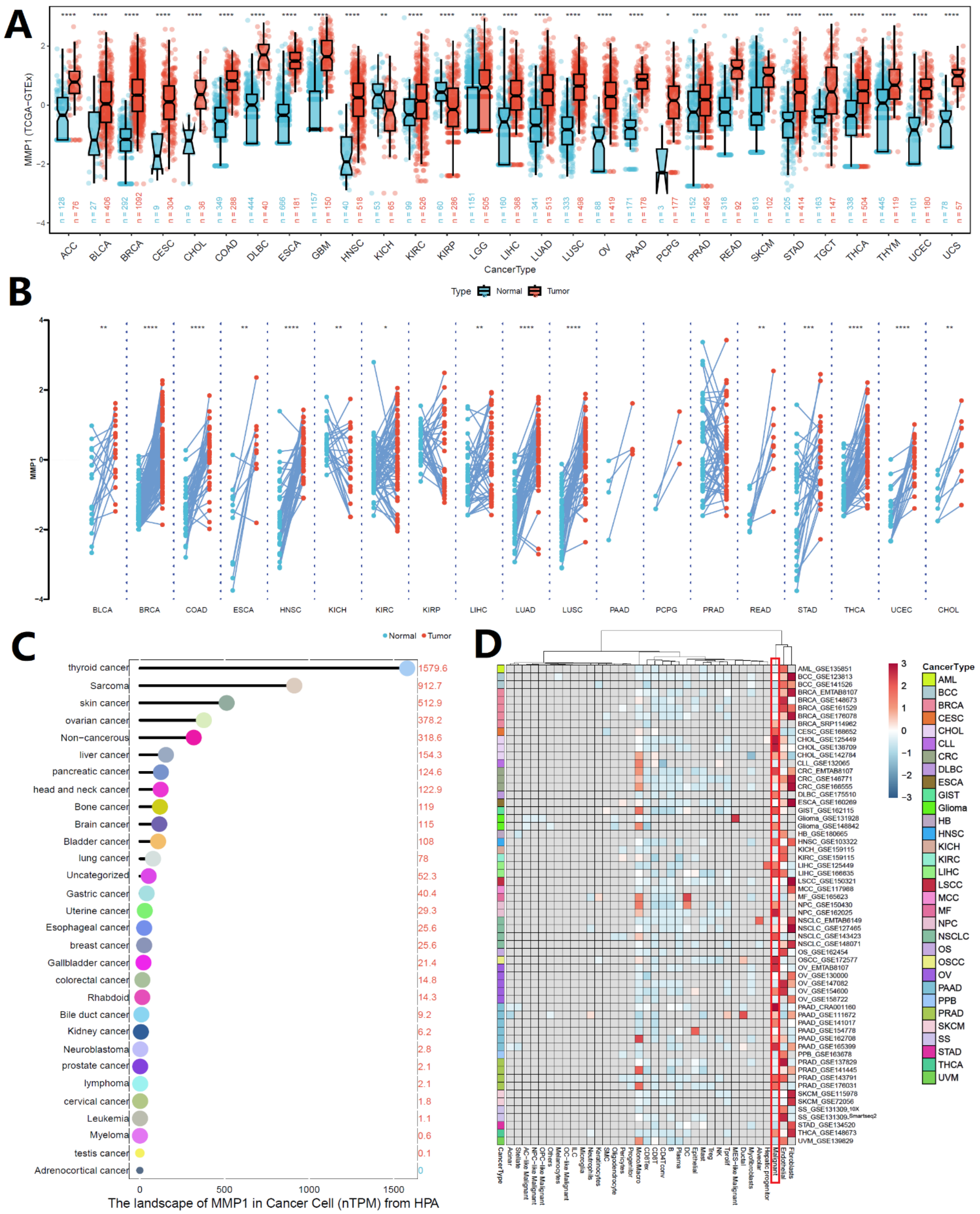

Fig. S1. Pan-cancer analysis of MMP1 expression

(A) Differential expression of MMP1 in tumor tissues compared to normal tissues.

(B) Differential expression of MMP1 between tumor tissues and paired adjacent normal tissues.

(C) MMP1 expression in various tumor cells based on the HPA database.

(D) MMP1 expression in various tumor single-cell database.
